# Progress of the Surgery of the Vermiform Appendix

**Published:** 1904-02-13

**Authors:** 


### PROGRESS OF THE SURGERY OF THE VERMIFORM APPENDIX.

**Appendicitis.**—Eccles[1] thinks that far too little attention is paid to previous attacks of appendicitis by the medical officers to life insurance companies. Against loss by death from a primary attack no insurance company can be protected, but any history of previous attacks should be inquired for. If, however, the appendix has been removed, and six months have elapsed since the operation, ordinary rates only should be charged. He has succeeded in collecting 14 instances of undoubted primary carcinoma, and three of primary sarcoma of the appendix. Moullin[2] discusses the significance of pain and tenderness in cases of inflammation of the appendix. He points out that inflammation of the parietal peritoneum or of the post-peritoneal cellular tissue, whether due to an irritant acting directly upon the serous surfaces or to absorption from some comparatively distant source of infection, naturally makes the parts that are sensitive already tenfold more so. The slightest traction becomes almost intolerable. On the other hand, inflammation has no effect whatever upon the visceral layer. The wall of the bowel, whether inflamed or not is quite insensitive. It has no sensory nerves. It therefore follows that in the absence of other mechanical stimuli the cause of abdominal pain and colic is either pressure or traction upon the branches of the spinal nerves, which are distributed mainly if not entirely to the abdominal walls, and that the intensity of the pain, so far as the individual is concerned, depends upon the degree of traction or pressure on the one hand and upon the condition of the surrounding tissues, as regards inflammation upon the other. It follows, moreover, that in acute inflammation of the appendix :—1. Absence of pain is no indication that the most serious mischief is not going on. 2. The initial pain of acute inflammation of the appendix which is so commonly referred to the umbilicus is due to the peristaltic action of the cæcum or of the appendix dragging upon the attachment of the peritoneum to the abdominal wall. This is especially likely to occur when the appendix is fixed by adhesions or the mesentery is short or twisted upon itself. 3. The cessation of this umbilical pain without improvement in the other symptoms is due to cessation of the peristalsis caused by the inflammation having spread to the muscular coat of the bowel. When this occurs the wall of the bowel is no longer able to contract, and this source of pain disappears. 4. The development of local pain, which as a rule precedes by some short time the cessation of the umbilical pain, means that the inflammation has spread from the appendix to the parietal peritoneum or to the post-peritoneal cellular tissue. These are the only structures concerned (at this period of the disease) which are supplied with nerves capable of transmitting the sensation of pain. Absence of local pain does not indicate anything. Severe pain is of serious import, as it implies either wide extent or great severity of inflammation. Barnard[3] draws attention to the following facts which he considers must of necessity guide us in our treatment :—(1) The attack mortality of all cases of appendicitis, slight or severe, is estimated by Treves to be about 5 per cent. (2) The attack mortality of severe cases derived from the records of a large number of hospital cases which were not operated on, is about 20 per cent. (3) There is no doubt that first attacks are much more fatal, and that they are especially fatal in children. Their high mortality is due to the fact that nearly all abscesses and general peritonitis cases arise in the first attack. A line of treatment which allows all severe attacks to run their course, and is content to stop relapses by an operation during the quiescent stages, can at best only claim to reduce the general mortality 7 per cent., and passively acquiesces in a mortality of 20 per cent. in these severe cases. (4) About a quarter to a third of all cases relapse within a few years. (5) It is estimated that these relapses add about 7 per cent. to the general mortality of all severe unoperated cases. This mortality is of importance when considering the question of an interim operation. (6) Fulminating appendicitis, by which we understand cases of appendicitis which die of general peritonitis or septicæmia within three days of the onset of the attack, forms about 1 per cent. of mild and 2 per cent. of severe cases. It will be noticed how small this percentage is, and therefore it cannot be used, as it has been, as an argument in favour of promiscuous operation, and certainly not for universal operating in cases after the third day. (7) Probably the most important point in the question of operation is the surgeon, whether he is skilled in abdominal work and has had a large experience of appendicitis, or whether, on the other hand, he is inexperienced, and in the nature of things is not likely to have gained any special knowledge of surgical interference in this disease. (8) The interim operation has a mortality in competent hands of less than 1 per cent., but this is not a mortality which can be counted on by every ordinary operating surgeon. (9) The mortality of operation in the first 24 hours of first attacks is probably 5 to 10 per cent. in the hands of competent surgeons. (10) Operations on the fourth and fifth days have a very severe mortality, 40 per cent., according to Fowler. (11) Operations on localised abscesses give excellent results. (12) Operations on cases of really general suppurative peritonitis give deplorable results. In the treatment of general suppurative peritonitis he employs massive saline subcutaneous transfusions.[4] The reservoir is placed about one foot above the patient. If a greater height than this is employed the distension gives rise to considerable pain. The transfusion is made into the thighs, and with this elevation two pints of saline fluid will flow into the subcutaneous tissues in an hour through the two needles. A full-grown man can safely take 15 pints in the first 24 hours after the operation. The transfusion is repeated day by day as occasion demands, unless the vomiting ceases and the patient can drink freely, or the rectum will absorb a pint six times a day. This saline method is based on many sound reasons. In the first place the body is very short of fluid, and it is a well-known fact in biology that all protoplasm in order to perform its functions well, must be distended with water. In the second place these pressure transfusions alter the relation between the vascular system and the pus in the peritoneum. In the third place the transfusion dilutes the toxins circulating in the blood, and probably in this way diminishes the virulence of their action on nervous tissues, on heart muscle, and on the other tissues of the body. In the last place this flood of saline fluid passing through the body carries out with it, especially through the kidneys, these toxins in a very dilute form, and with the least possible damage to the renal epithelium. Sherren,[5] writing on the occurrence and significance of cutaneous hyperalgesia in appendicitis forms the following conclusions: (1) cutaneous hyperalgesia is probably present at some time during all first attacks of appendicitis, except, perhaps, in the fulminating type, and depends upon tension within the appendix; (2) it may be absent in attacks after the first, if the first attack was of sufficient severity to destroy nerve tissue in the walls of the appendix; (3) when present in attacks subsequent to the first it often persists long after all other signs of the disease have gone, owing to the tension within the appendix being kept up by the presence of a stricture; (4) it gradually disappears during convalescence as the other signs of the disease clear up; (5) disappearance of cutaneous hyperalgesia without improvement in the general condition of the patient, is a sign of perforation or gangrene of the appendix, and should be a signal for immediate operation; (6) the presence of cutaneous hyperalgesia is no contra-indication to operation. Abscesses may form, and general peritonitis may develop while it is present; (7) its absence, on the other hand, is of great importance. Absence of cutaneous hyperalgesia, the patient coming under observation early in the first attack of appendicitis, is a sign of gangrene of the appendix unless the case is obviously a mild one, and the patient is rapidly getting well; (8) cutaneous hyperalgesia is, as a rule, absent in cases of abscess of the appendix; (9) the age of the patient and the position of the appendix have no influence upon the cutaneous hyperalgesia; (10) it is occasionally of use as an aid to the diagnosis of appendicitis. Weir[6] records some interesting cases of unusual occurrence.

[1] Lancet, April 11, 1903, p. 1042. [2] Ibid, August 22, 1903, p. 514. [3] Clinical Journal, September 16, 1903, p. 342. [4] Ibid., September 30, 1903, p. 381. [5] Lancet, September 19, 1903, p. 816. [6] Med. Rec., New York, May 23, 1903, p. 801.

